# Age of onset determines intrinsic functional brain architecture in Friedreich ataxia

**DOI:** 10.1002/acn3.50966

**Published:** 2019-12-18

**Authors:** Gilles Naeije, Vincent Wens, Nicolas Coquelet, Martin Sjøgård, Serge Goldman, Massimo Pandolfo, Xavier P. De Tiège

**Affiliations:** ^1^ Laboratoire de Cartographie fonctionnelle du Cerveau ULB Neuroscience Institute (UNI) Université libre de Bruxelles (ULB) Brussels Belgium; ^2^ Department of Neurology CUB Hôpital Erasme Université libre de Bruxelles (ULB) Brussels Belgium; ^3^ Department of Functional Neuroimaging Service of Nuclear Medicine CUB Hôpital Erasme Université libre de Bruxelles (ULB) Brussels Belgium

## Abstract

**Objective:**

Friedreich ataxia (FRDA) is the commonest hereditary ataxia in Caucasians. Most patients are homozygous for expanded GAA triplet repeats in the first intron of the frataxin (*FXN*) gene, involved in mitochondrial iron metabolism. Here, we used magnetoencephalography (MEG) to characterize the main determinants of FRDA‐related changes in intrinsic functional brain architecture.

**Methods:**

Five minutes of MEG signals were recorded at rest from 18 right‐handed FRDA patients (mean age 27 years, 9 females; mean SARA score: 21.4) and matched healthy individuals. The MEG connectome was estimated as resting‐state functional connectivity (rsFC) matrices involving 37 nodes from six major resting state networks and the cerebellum. Source‐level rsFC maps were computed using leakage‐corrected broad‐band (3–40 Hz) envelope correlations. Post hoc median‐split was used to contrast rsFC in FRDA patients with different clinical characteristics. Nonparametric permutations and Spearman rank correlation test were used for statistics.

**Results:**

High rank correlation levels were found between rsFC and age of symptoms onset in FRDA mostly between the ventral attention, the default‐mode, and the cerebellar networks; patients with higher rsFC developing symptoms at an older age. Increased rsFC was found in FRDA with later age of symptoms onset compared to healthy subjects. No correlations were found between rsFC and other clinical parameters.

**Conclusion:**

Age of symptoms onset is a major determinant of FRDA patients' intrinsic functional brain architecture. Higher rsFC in FRDA patients with later age of symptoms onset supports compensatory mechanisms for FRDA‐related neural network dysfunction and position neuromagnetic rsFC as potential marker of FRDA neural reserve.

## Introduction

Friedreich ataxia (FRDA) is the most common autosomal recessive ataxia in Caucasians.^1^ It is mainly caused by homozygous intronic GAA triplet expansions in the frataxin (*FXN*) gene,^2^ which repress *FXN* expression via an epigenetic mechanism. The length of the shorter GAA repeat (GAA1) explains 30–50% of the variability in age of symptoms onset (mean: 12–16 years, range: 1–52 years),^3^, ^4^ but fails to accurately predict FRDA onset, clinical evolution or severity in individual cases.

FRDA is characterized by early atrophy of large neurons in dorsal root ganglia (DRGs) and of the posterior columns in the spinal cord, followed by progressive atrophy of the dentate nuclei in the cerebellum and of efferent fibers in the superior cerebellar pedunculus.^5^ This leads to a “tabeto‐cerebellar” ataxia ending in gait loss after 10–15 years.^6^ FRDA is further complicated by variable alterations in visual, auditory, pyramidal, and cognitive systems.^7^

Little is known about how spinal, cerebellar, and cerebral pathology interact along the course of FRDA and about the key determinants of these interactions. The study of resting state (i.e., in the absence of any explicit task) functional connectivity (rsFC) allows to noninvasively characterize brain interactions in FRDA using a task and performance bias‐free approach. Previous functional Magnetic Resonance Imaging (fMRI) studies performed in autosomal dominant spinocerebellar ataxias (SCAs, e.g., SCAs 2, 6, and 7) demonstrated significant changes in cerebello‐cortical and cortico‐cortical rsFC that correlated with to some extent with various clinical variables.^8^, ^9^, ^10^, ^11^, ^12^ However, the pattern of cerebellar pathology in these SCAs is different from FRDA, with the cerebellar cortex being primarily affected. As a consequence, at least in their early stages, these conditions are characterized by excessive, uninhibited deep cerebellar nuclei output^13^ rather than by impaired dentate output as in FRDA. A single fMRI study performed in FRDA demonstrated increased rsFC between brain areas potentially involved in specific cognitive functions, and decreased rsFC of cerebello‐prefrontal connections.^14^ Increases in rsFC were considered to reflect compensatory mechanisms, as they tended to correlate with some neuropsychological scores.

Although fMRI is the most commonly used noninvasive technique to investigate rsFC in humans, this modality might be inappropriate in FRDA. First, mitochondrial dysfunction, intrinsic to the disease,^15^, ^16^, ^17^ might lead to neurovascular uncoupling. Second, the high prevalence of abnormal carbohydrate metabolism in FRDA represents an additional risk of neurovascular uncoupling.^18^ Magnetoencephalography (MEG) is free of these limitations as it directly records neural activity. We therefore utilized MEG in FRDA patients to characterize changes within and between the major brain networks that are typically identified at rest, the so‐called “resting‐state networks” (RSNs). With the goal of gaining insights into the key determinants of FRDA patients’ intrinsic functional brain architecture, we first compared the functional brain connectivity of FRDA patients with that of matched healthy subjects. We then searched for correlation between rsFC changes, genetic, and clinical parameters (i.e., the size of GAA1 triplet expansion, age of symptoms onset, disease duration, and severity of clinical symptoms).

## Methods

The signal processing methods used in this study are derived from^19^.

### Participants

Eighteen FRDA patients (9 females, see Table 1 for additional characteristics) and matched healthy subjects (9 females; mean age: 29 yrs, range: 10–47 years) without any history of neurologic or psychiatric disease were studied after written informed consent. The study had prior approval by the CUB Hôpital Erasme Ethics Committee. Sixteen of those subjects participated in previous studies from our group investigating the pathophysiology of sensory deficits in FRDA.^20^, ^21^

**Table 1:** Characteristics of the included FRDA patients.

Age (mean, [range], years)	27 [9–46]
SARA (median, [range])	21 [4.5–32]
Age of symptoms onset (median, [range], years)	11 [4–30]
Disease duration (median, [range]; years)	12.5 [4–38]
GAA1 (median, [range])	670 [280–1000]

SARA, score on the Scale for the Assessment and Rating of Ataxia; GAA1, size of GAA1 triplet expansion on the shortest allele.

### MEG data acquisition and structural MRI

Neuromagnetic activity was recorded at rest (5 min, eyes opened, fixation cross, online band‐pass filter: 0.1–330 Hz, sampling frequency: 1 kHz) with a 306‐channel whole‐scalp‐covering MEG system (Vectorview, MEGIN, 12 FRDA patients, seven healthy subjects; Triux, MEGIN, six FRDA patients, eleven healthy subjects; Croton Healthcare, Helsinki, Finland) installed in a lightweight magnetically shielded room (Maxshield, MEGIN, Croton Healthcare, Helsinki, Finland). Four head‐tracking coils continuously monitored subjects’ head position inside the MEG helmet. The location of the coils and at least 200 head‐surface points were determined with respect to anatomical fiducials with an electromagnetic tracker (Fastrak, Polhemus, Colchester, VT). Participants’ high‐resolution 3D‐T1 weighted cerebral MRI were acquired on a 1.5T MRI scanner (Intera, Philips, The Netherlands).

### Data preprocessing

The signal space separation method was first applied offline to continuous MEG data to reduce external magnetic interferences and correct for head movements.^22^ Then, ocular, cardiac, and system artifacts were identified and regressed out from raw MEG signals using an independent component analysis (FastICA algorithm on band‐passed (0.1–45 Hz) data, dimension reduction to 30 components, hyperbolic tangent nonlinearity).

To investigate wide‐band rsFC rather than band‐specific rsFC,^23^ the cleaned MEG data were subsequently broad‐band (3–40 Hz) filtered as done in^23^, who demonstrated significant neuromagnetic rsFC changes in patients with amyotrophic lateral sclerosis. Band‐limited power time courses were then obtained by taking the Hilbert envelope of the broad‐band signal, low‐pass filtered at 1 Hz.

### Source reconstruction

Participants’ MRI were anatomically segmented using FreeSurfer (Martinos Center for Biomedical Imaging, MA). MEG and MRI coordinate systems were manually co‐registered. Then, a 5‐mm regular grid of dipole locations was built in the Montreal Neurological Institute (MNI) template MRI and nonlinearly deformed onto each participant’s MRI with Statistical Parametric Mapping (SPM8, Wellcome Trust Centre for Neuroimaging, London, UK). The MEG forward model associated with this source space was finally computed using the one‐layer Boundary Element Method implemented in the MNE‐C suite (Martinos Centre for Biomedical Imaging, MA).

Minimum Norm Estimation (MNE) based on planar gradiometers only^24^ was then applied to estimate broad‐band source activity (noise covariance taken from 5 min band‐passed (3–40 Hz) empty‐room data; regularization parameter fixed with consistency condition from^25^; depth bias correction via noise normalization of^26^). Three‐dimensional dipole time series were projected on their direction of maximum variance. The power time course of the resulting source signals were obtained by taking their Hilbert envelope and further low‐pass filtering at 1 Hz.^27^

### Functional connectivity estimation

**Figure 1:**
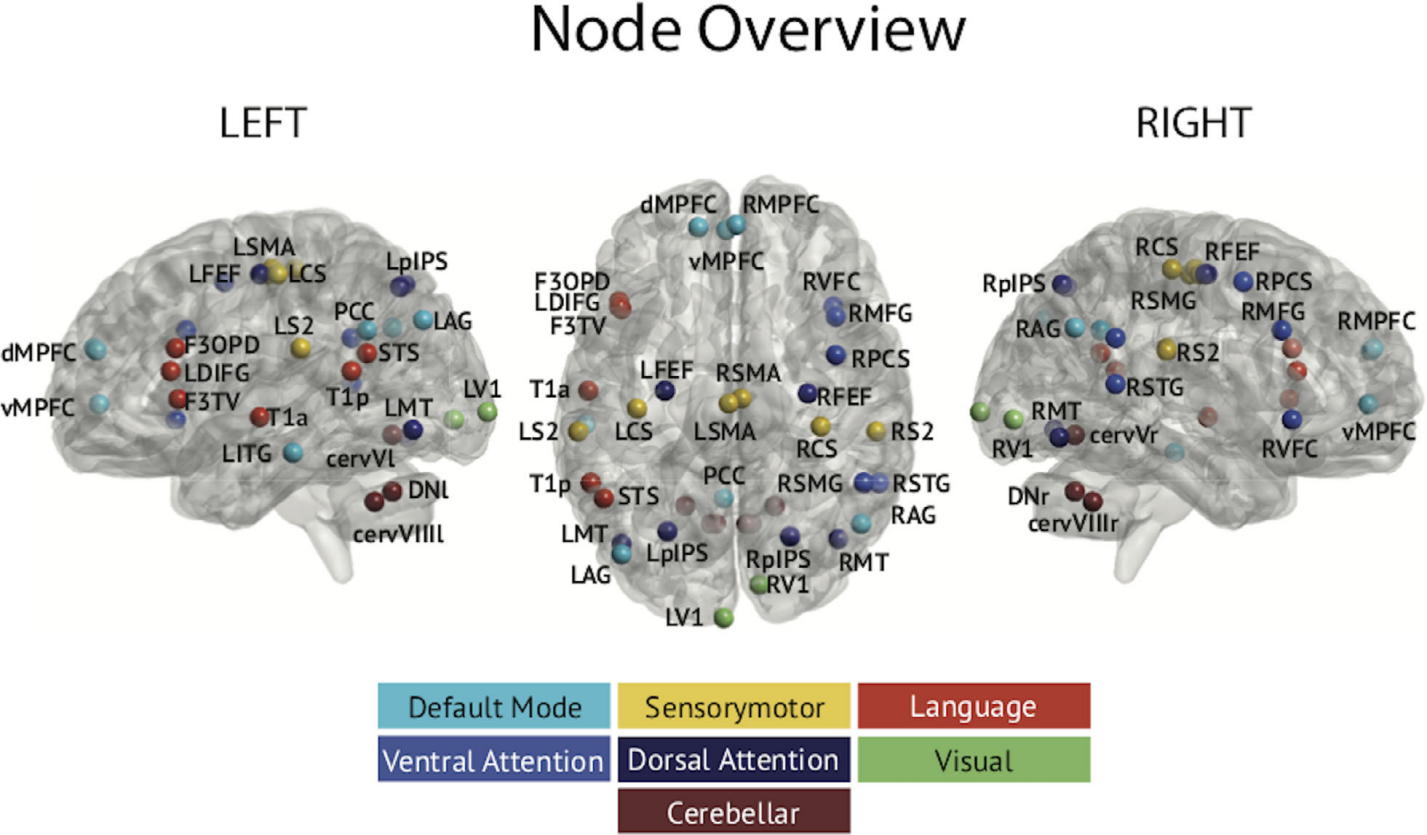
Overview of nodes location rendered on a glass brain model. Brain is viewed from the left side, from above, and from right side respectively. Dorsal attention, dorsal attention network nodes; Ventral attention, ventral attention network nodes; Default mode, default mode network nodes. Sensorymotor, motor network nodes; Language, language network nodes. Coordinates and labels abbreviations may be found in 57. Cerebellar, Cerebellar networks node. Cerebellar lobule V (cerV), Cerebellar lobule VIII (cervVIII), dentate nuclei (DN).

A connectome approach with slow envelope (i.e., low‐passed at 1 Hz) correlation was used for rsFC estimation. This measure was chosen as it provides greater repeatability than other functional connectivity indices^28^ and because it allows uncovering fMRI RSNs.^27^, ^29^ Envelope correlation was estimated with geometric correction for spatial leakage effects^25^ among 31 neocortical locations extracted from six well‐known RSNs: default mode network (DMN), dorsal attentional network (DAN), ventral attentional network (VAN), visual network (VISN), sensorimotor network (SMN), language network (LAN).^30^ Six extra nodes corresponding to a cerebellar network (dentate nuclei, anterior, and posterior lobes; MNI coordinates derived from^31^) were added. Figure 1 illustrates the location of the 37 RSN nodes. The resulting rsFC matrices were symmetrized.^19^ Signal power at the 37 nodes was also estimated to control for power‐induced effects in rsFC changes. Significance of the group‐level contrast (i.e., FRDA patients vs. healthy subjects) was appraised using nonparametric permutation tests of power or rsFC differences (10^6^ permutations of group‐membership label) at *P* < 0.05 with the multiple comparison problem (37 nodes or 666 connections) controlled by Bonferroni correction for the number of degrees of freedom (power: 20, rsFC: 182) as in^19^. Corrected *P* values were obtained by multiplication of the uncorrected *P* values by the Bonferroni factor.

Previous studies from our group showed that pooling datasets from different MEG systems do not alter results in both evoked responses or rsFC investigations.^20^, ^32^ Yet, to rule out an effect induced by the MEG system on rsFC in this study, we also tested for correlation between rsFC connectivity and MEG system types (i.e., Vectorview vs. Triux).

### Node connectivity strength

Groups were also contrasted regarding “*node strength*,”^33^ that is, the average rsFC value of all connections to each node. This measure determines how strongly connected a node is to the rest of the brain. Contrast significance testing was similar than for node power in 2.5.

### Global connectivity

Mean rsFC across all connections was computed to measure how strongly the brain is globally connected. Group comparison was assessed statistically using Wilcoxon Rank Sum test.

### Correlation analyses

Values of rsFC, node strength, and node power in FRDA patients were independently correlated with the size of GAA1 triplet expansion, the age of symptoms onset, the SARA score, and disease duration using Spearman rank correlation. The multiple comparison problem across connections or nodes was controlled as described above.

### Data availability statement

De‐identified participants’ data will be shared as well as study protocol and statistical analyses upon request.

## Results

### Contrast analyses between FRDA patients and healthy subjects

**Figure 2:**
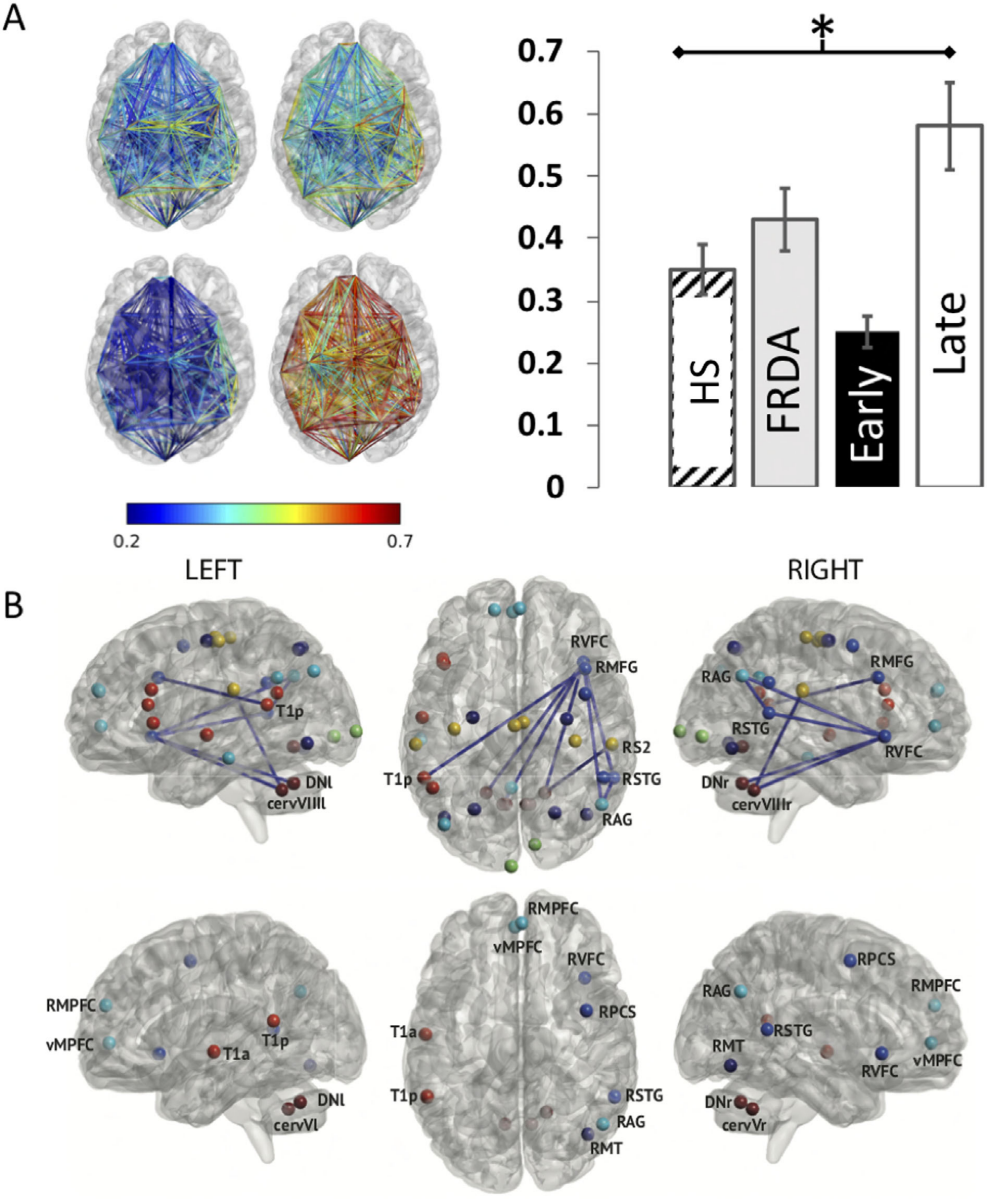
(A) Top. Mean rsFC between nodes in healthy subjects (Left) and FRDA patients (Right). Left, Bottom. Mean rsFC between nodes in FRDA with early (Left) and late (Right) age of symptoms onset. Right. Histogram showing mean rsFC in healthy subjects (HS, hatched), whole group of FRDA patients (gray), patients with early (black), and late (white) age of symptoms. *above thick line = corrected *P* < 0.001. (B) Top. Connections with rsFC levels significantly different in FRDA patients with late age of symptoms onset compared with healthy subjects. Bottom. Nodes with significantly different mean node strength in FRDA patients with late age of symptoms onset compared with healthy subjects. Brain are viewed from the left side (Left), above (Middle), and right side (Right) respectively.

Figure 2A illustrates the results of rsFC contrasts. No significant difference in power, rsFC, node strength, and global connectivity was found between FRDA patients and healthy subjects.

### Correlations with clinical parameters in FRDA patients

**Figure 3:**
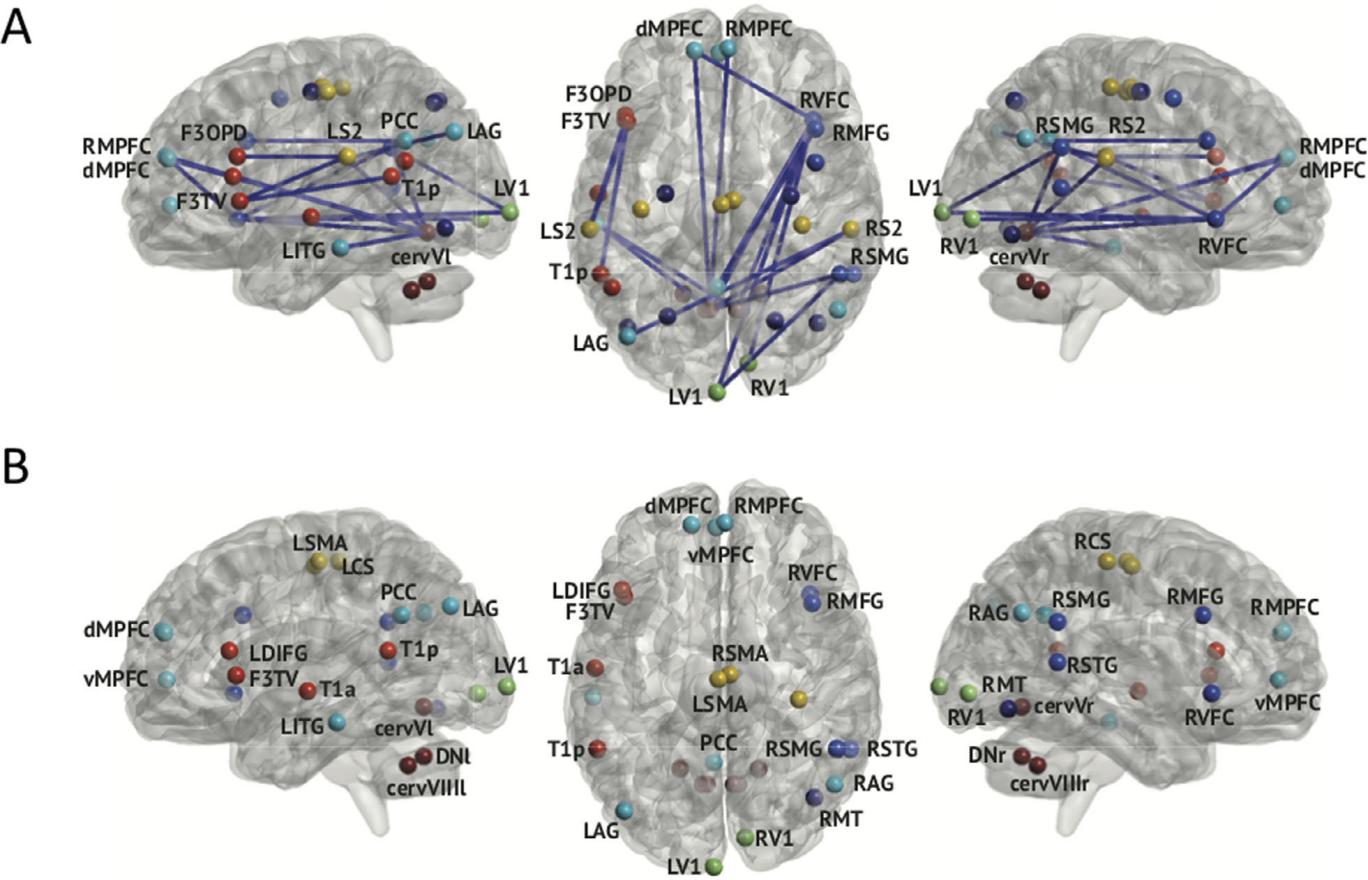
(A) Connections with rsFC levels that significantly correlate with age of symptoms onset in FRDA patients. (B) Nodes whose weight significantly correlate with age of symptoms onset in FRDA patients. Brain is viewed from the left side (Left), above (Middle), and right side (Right) respectively.

Figure 3 illustrates results of the correlation analysis between rsFC and clinical parameters in FRDA patients. Significant positive correlations were found in FRDA patients only between the age of symptoms onset and 18 (mainly) cross‐network connections (Fig. 3A) as well as with the node strength of 27 nodes of 37 (Fig. 3B). Tables 2 (rsFC) and 3 (node strength) detail the correlations between the age of symptoms onset and rsFC/node strength in FRDA patients. No other correlation was found significant with other clinical parameters or with the MEG system type (supplementary material).

**Table 2:** Correlation between age of symptoms onset and rsFC.

Connection	Corresponding RSNs	*R*	Corrected *P*
RMFG‐PCC	VAN‐DMN	0.80	0.009
RVFC‐PCC	VAN‐DMN	0.77	0.026
RVFC‐dMPFC	VAN‐DMN	0.76	0.026
RVFC‐LV1	VAN‐Visual	0.81	0.007
RVFC‐RV1	VAN‐Visual	0.75	0.047
RVFC‐RV1	VAN‐Visual	0.76	0.042
LAG‐RS2	DMN‐Sensorimotor	0.76	0.041
LS2‐F3OPD	Sensorimotor‐Language	0.76	0.041
LS2‐F3TV	Sensorimotor‐Language	0.80	0.019
F3TV‐T1p	Language‐Language	0.80	0.011
RSMG‐cervVl	VAN‐Cerebellar	0.86	0.0007
dMPFC‐cervVl	DMN‐Cerebellar	0.76	0.037
RMPFC‐cervVl	DMN‐Cerebellar	0.76	0.039
LITG‐cervVl	DMN‐Cerebellar	0.78	0.021
RS2‐cervVl	Sensorimotor‐Cerebellar	0.76	0.033
RVFC‐cerVr	VAN‐Cerebellar	0.79	0.015
LITG‐cerVr	DMN‐Cerebellar	0.76	0.041

**Table 3:** Correlation between age of symptoms onset and node strength.

Node	Corresponding RSNs	*R*	Corrected *P*
RMT	DAN	0.66	0.046
RMFG	VAN	0.69	0.028
RSMG	VAN	0.72	0.013
RSTG	VAN	0.72	0.013
RVFC	VAN	0.68	0.033
LAG	DMN	0.69	0.028
RAG	DMN	0.72	0.013
PCC	DMN	0.74	0.008
vMPFC	DMN	0.67	0.041
dMPFC	DMN	0.70	0.020
RMPFC	DMN	0.74	0.008
LITG	DMN	0.66	0.046
LV1	Visual	0.78	0.002
RV1	Visual	0.77	0.002
RCS	Sensorimotor	0.69	0.028
LSMA	Sensorimotor	0.74	0.007
RSMA	Sensorimotor	0.74	0.007
LDIFG	Sensorimotor	0.73	0.010
T1a	Language	0.68	0.034
F3TV	Language	0.73	0.010
T1p	Language	0.77	0.003
DNl	Cerebellar	0.72	0.011
DNr	Cerebellar	0.70	0.023
cervVl	Cerebellar	0.75	0.006
cerVr	Cerebellar	0.74	0.008
cervVIIIl	Cerebellar	0.72	0.012
cervVIIIr	Cerebellar	0.70	0.019

### Median split analysis

To further characterize the above results, we used a post hoc median split on age of symptoms onset (i.e., FRDA subjects split into 2 subgroups, <11 years and >11 years) similarly to^34^ to search for significant differences in rsFC, node strength, and global connectivity between those two subgroups and healthy subjects. This post hoc analysis was used to determine if patients with early (<11 years) or late (i.e., >11 years) age of symptoms onset presented power, rsFC, or node strength levels that were significantly different from healthy subjects. These contrasts were analyzed using the same method as in 2.5–7.

Analyses disclosed significantly higher rsFC, node strength, and global connectivity in FRDA patients with a later age of symptoms onset (>11 years; mean rsFC values ±standard deviation: 0.60 ± 0.23; rsFC variation coefficient: 0.38) compared with healthy subjects (0.35 ± 0.13; 0.37), but no power differences (Fig. 2B). No significant difference was observed between FRDA patients with an earlier age of symptoms onset (<11 years, 0.25 ± 0.05; 0.2) compared with healthy subjects (0.35 ± 0.13; 0.37).

Tables 4 and 5 detail the connections with higher rsFC and the nodes with higher node strength in FRDA patients with later age of symptoms onset compared with healthy subjects.

**Table 4:** Difference in rsFC between healthy subjects and FRDA patients with an age of symptoms onset >11 years.

Connection	Corresponding RSNs	Corrected *P*
RSTG,RVFC	VAN‐VAN	0.007
RSTG,RAG	VAN‐DMN	0.040
RVFC,RAG	VAN‐DMN	0.049
RAG,vMPFC	DMN‐DMN	0.030
RAG,RMPFC	DMN‐DMN	0.023
RMFG,T1p	VAN‐Language	0.038
RVFC,DNl	VAN‐Cerebellar	0.034
RVFC,DNr	VAN‐Cerebellar	0.020
RS2,DNr	Sensorimotor‐Cerebellar	0.023
RVFC,cervVIIIl	VAN‐Cerebellar	0.007

**Table 5:** Difference in node strength between healthy subjects and FRDA patients with an age of symptoms onset >11 years.

Node	Corresponding RSNs	Corrected *P*
RMT	DAN	0.043
RPCS	VAN	0.043
RSTG	VAN	0.022
RVFC	VAN	0.017
RAG	DMN	0.037
vMPFC	DMN	0.018
RMPFC	VAN	0.013
T1a	Language	0.037
T1p	Language	0.008
DNr	Cerebellar	0.010
cervVIIIr	Cerebellar	0.021
cervVIIIl	Cerebellar	0.028

## Discussion

This MEG study demonstrates that the age of symptoms onset is a major determinant of the intrinsic functional brain architecture in FRDA patients. Indeed, it shows that (1) the strength of some cross‐network interactions between major RSNs (mainly between the VAN, the DMN, and the cerebellar network) as well as node strength for a large number of the studied RSNs correlate with the age at symptoms onset, and that (2) FRDA patients with later (i.e., >11 years) age of symptoms onset have higher rsFC, node strength, and global connectivity compared with matched healthy subjects.

At the group level, we failed to find any significant difference in power, rsFC, node strength or global connectivity between FRDA patients and healthy subjects. This can potentially be explained by three main factors. First, the clinical spectrum of the included FRDA patients covered a wide range of severity and disability stages, leading to a highly clinically heterogeneous group. This was reflected by the broad range of SARA scores, from 4.5 (slight disability) to >30 (severe disability, wheelchair bound), age of symptoms onset (4–30 years), and size of GAA1 triplet expansion (280–1000). Second, we did not assume any a priori when we compared power, rsFC, and node strength across 37 seeds between FRDA patients and healthy subjects*.* This implied the use of appropriate statistical thresholding to correct for multiple comparisons. Third, the use of two different MEG systems could have biased the results. Yet, this is unlikely as there was no correlation between MEG system type and rsFC in our dataset, which corroborates previous studies from our group that found no effect of MEG system type when data were pooled.^32^, ^35^

In FRDA patients, we found that age of symptoms onset correlates with rsFC levels, mainly influencing cross‐network interactions involving the VAN, the DMN, and the cerebellum network. This was particularly evident at the right inferior and middle frontal gyri, which are both core to the VAN, and at the cerebellar nodes. Similar correlations between age of symptoms onset and node strength occurred in nodes belonging to the same networks.

The absence of correlation between rsFC and the size of GAA1 triplet expansion is not surprising because only 30–50% of the variability in age of symptoms onset is explained by this variable.^4^ Such limited correlation may be explained by the effect of genetic and nongenetic modifiers and by the somatic instability of the GAA1 triplet expansion, typically measured in peripheral blood mononuclear cells (PBMCs), with variable repeat lengths in different tissues, notably in the CNS.^36^, ^37^ Despite the often insidious onset of FRDA, age at symptoms onset more closely reflects the progression of pathology in individual patients and therefore it correlates more closely than to the size of GAA1 triplet expansion to brain rsFC.

On the basis of the correlation results and as done in a previous neuroimaging study in FRDA,^34^ we used a post hoc median split subgroup analysis to search for significant differences in rsFC between healthy subjects and more homogenous groups of FRDA patients. This post hoc analysis aimed at obtaining further insights into the influence of the age of symptoms onset on FRDA patients' brain rsFC. Patients who developed symptoms after age 11 showed increased rsFC connectivity and node strength compared with healthy subjects, as well as increased rsFC in 10 connections mainly involving the VAN, the DMN and the cerebellar network. No difference was observed between patients with earlier (<11 years) age of symptoms onset and healthy subjects.

Decreased rsFC in brain disorders is classically interpreted as reflecting disease‐induced disruption of functional integration,^38^, ^39^ whereas increased rsFC is considered as a compensatory mechanism, either promoting functional recovery by helping structurally damaged brain areas to remain functional,^9^ for example, after stroke,^40^, ^41^ or to functionally compensate for pathological alterations occurring in distant brain areas.^33^, ^42^, ^43^ Therefore, the observed increase in rsFC in FRDA patients with later age of symptoms onset may represent a compensatory mechanism. The lack of correlation between rsFC and disease duration/severity observed in the present MEG study potentially suggests the occurrence of a “*threshold effect*,” that is, compensatory mechanisms as indexed by increased neuromagnetic rsFC are required to delay the age of symptoms onset, and when they cannot take place or are overwhelmed (i.e., rsFC values similar to those of healthy subjects), patients become symptomatic. However, we included a limited and clinically heterogeneous number of FRDA patients, which may have clouded correlations with disease duration/severity that would have potentially arisen with larger and clinically more homogenous group of patients.

Our findings are partly in line with a previous resting‐state fMRI study in FRDA patients,^14^ which demonstrated increased rsFC between brain areas potentially involved in specific cognitive functions. A trend for correlation of rsFC levels with some neuropsychological test scores was indeed observed, although not significant after correction for multiple comparisons. This was interpreted as evidence for a compensatory mechanisms in response to the multi‐system neural degeneration characterizing FRDA.^14^ The same study also showed decreased rsFC between the cerebellum and the mesial frontal cortex, interpreted as reflecting pathology in cerebello‐cortical pathways. Crucially, in that study, there was no correlation between brain rsFC levels and SARA score, the size of GAA1 triplet expansion, or disease duration, whereas correlation with age of symptoms onset was not assessed.

Previous structural MRI and diffusion tensor imaging (DTI) studies found widespread white matter connectivity disruptions involving both cerebello‐cerebral and some cerebro‐cerebral connections,^44^, ^45^ with a thinning of neuronal tracks’ density correlating, as in our study, with the age of symptoms onset.^44^, ^45^ These findings might suggest that the age of symptoms onset in FRDA differentially affects the structural and the functional brain connectivity, but further multimodal studies are needed to confirm such hypothesis.

Overall, this study demonstrates a strong relationship between the age of symptoms onset in FRDA patients and rsFC, likely to be driven by compensatory mechanisms in response to the multisystem neural degeneration characterizing this disease that allow patients to develop of symptoms at a later age. FRDA patients may present subtle signs of proprioceptive loss, such as loss of tendon reflexes and a Romberg sign, before they become frankly symptomatic. However, they become overtly ataxic only when cerebellar symptoms do appear. Those observations are corroborated by pathological and functional studies that found dorsal root ganglia and proprioceptive pathways alterations already pronounced early in the course of FRDA and stable along time.^20^, ^46^ In contrast, cerebellar pathology can only be detected after several years of disease progression and is associated with dentate nucleus progressive atrophy and alterations of cerebello‐cerebral and cerebro‐cerebral connections.^44^, ^45^ Therefore, the higher rsFC observed in this study in patients with a late age of symptoms onset probably accounts for the neural mechanisms that compensate the progressive cerebellar and cortical dysfunction associated with the neurological deterioration. The prominent involvement of VAN cross‐network interactions may underlie ataxia symptoms compensation because this network is involved in detecting unexpected but behaviorally relevant events in the environment and is essential to spatial attention.^47^ Ataxic patients need to rely more on attentional mechanisms to adapt to their environment, perform complex movement sequences, and compensate diminished afferent somatosensory information and cerebellar dysfunction.^48^, ^49^, ^50^ Congruent with this hypothesis, previous fMRI studies have demonstrated increased activations within the VAN during simple finger tasks^34^ in mildly affected FRDA patients.^34^ These findings further suggest the occurrence of initial early compensation within high‐level brain networks to mitigate neurological deficits caused by dysfunctional systems in neurodegenerative diseases. The cerebellum, through its connections with multiple cortical areas, plays an important role in perceptual processes (e.g., somatosensory change detection^51^) and high‐level cognitive functions such as language, memory, and executive functions (for reviews, see, e.g.,^52^) This explains why cerebellar dysfunction and atrophy in SCAs and atrophy of dentato‐thalamic tracts in FRDA^53^ lead to reduced cognitive processing speed, lower performance in complex visuospatial tasks, impaired executive functioning across tasks assessing cognitive flexibility and inhibition, as well as poorer ideas generation.^54^

Therapeutic approaches in FRDA investigate either improving mitochondrial function or the beneficial effects of increasing FXN levels in affected tissues. In different FRDA mouse models, gene therapy using injection of adeno‐associated virus expressing FXN either intrathecally or in peripheral blood led to increased FXN levels throughout the CNS as well as neurological symptoms improvement.^55^, ^56^ In future studies addressing the effects of such FXN‐restoring therapies in FRDA patients, neuromagnetic rsFC could potentially contribute to select the optimal time‐point to start the treatment and monitor their effects on FRDA patients' intrinsic functional brain architecture.

In summary, this MEG study demonstrates a close link between the age of symptoms onset and the intrinsic functional brain architecture of patients with FRDA, in particular with the strength of cross‐network interactions and the node strength of the cerebellum network, the VAN, and the DMN. It provides empirical findings supporting the existence of compensatory mechanisms or neural reserve in some FRDA patients to foster later (>11 years) age of symptoms onset. Considering the robustness of neuromagnetic rsFC relying on power envelope correlation at the individual level,^28^ this study paves the way for the use of MEG as a potential marker, for example, for sorting presymptomatic FRDA patients and the determination of optimal time‐points for therapeutic trials.

## Conflicts of Interest

Naeije reports no conflicts of interest. Coquelet reports no conflicts of interest. Sjogard reports no conflicts of interest. Wens reports no conflicts of interest. Goldman reports no conflicts of interest. Pandolfo reports no conflicts of interest. De Tiège reports no conflicts of interest.

## Authors’ Contributions

(Gilles Naeije; MD, PhD; Université libre de Bruxelles (ULB), Brussels, Belgium; Author): designed and conceptualized study; conducted the experiments; analyzed the data; wrote the manuscript; designed the Figures.

(Nicolas Coquelet; Université libre de Bruxelles (ULB), Brussels, Belgium; Author): analyzed the data; contributed to the writing of Methods.

(Martin Sjogard; Université libre de Bruxelles (ULB), Brussels, Belgium; Author): analyzed the data; designed the figures.

(Vincent Wens; PhD; Université libre de Bruxelles (ULB), Brussels, Belgium; Author): Analyzed the data; drafted the manuscript for intellectual content.

(Serge Goldman; MD, PhD; Université libre de Bruxelles (ULB), Brussels, Belgium; Author): Drafted the manuscript for intellectual content; provided input for research design and interpretation.

(Massimo Pandolfo; MD, PhD; Université libre de Bruxelles (ULB), Brussels, Belgium; Author): Designed and conceptualized the study; drafted the manuscript for intellectual content; provided input for research design and interpretation.

(Xavier De Tiège; MD, PhD; Université libre de Bruxelles (ULB), Brussels, Belgium; Author): Designed and conceptualized the study; wrote the manuscript; drafted the manuscript for intellectual content; provided input for research design and interpretation.

## Supporting information


**Figure S1.** Histograms showing the distribution of correlation coefficients and uncorrected *P*‐values for correlations between age of symptoms onset, the size of GAA1 triplet expansion, Age, SARA score, disease duration, and MEG system type.

**Table S1**. Illustrates the correlations between rsFC and MEG system, age, disease duration, GAA1, and SARA associated to its *P*‐value.
