# Supplementary figures and images for: Age of onset determines intrinsic functional brain architecture in Friedreich ataxia
Source: Ann Clin Transl Neurol. 2019 Dec 18;7(1):94–104. doi: 10.1002/acn3.50966 (PMC6952309; doi:10.1002/acn3.50966)

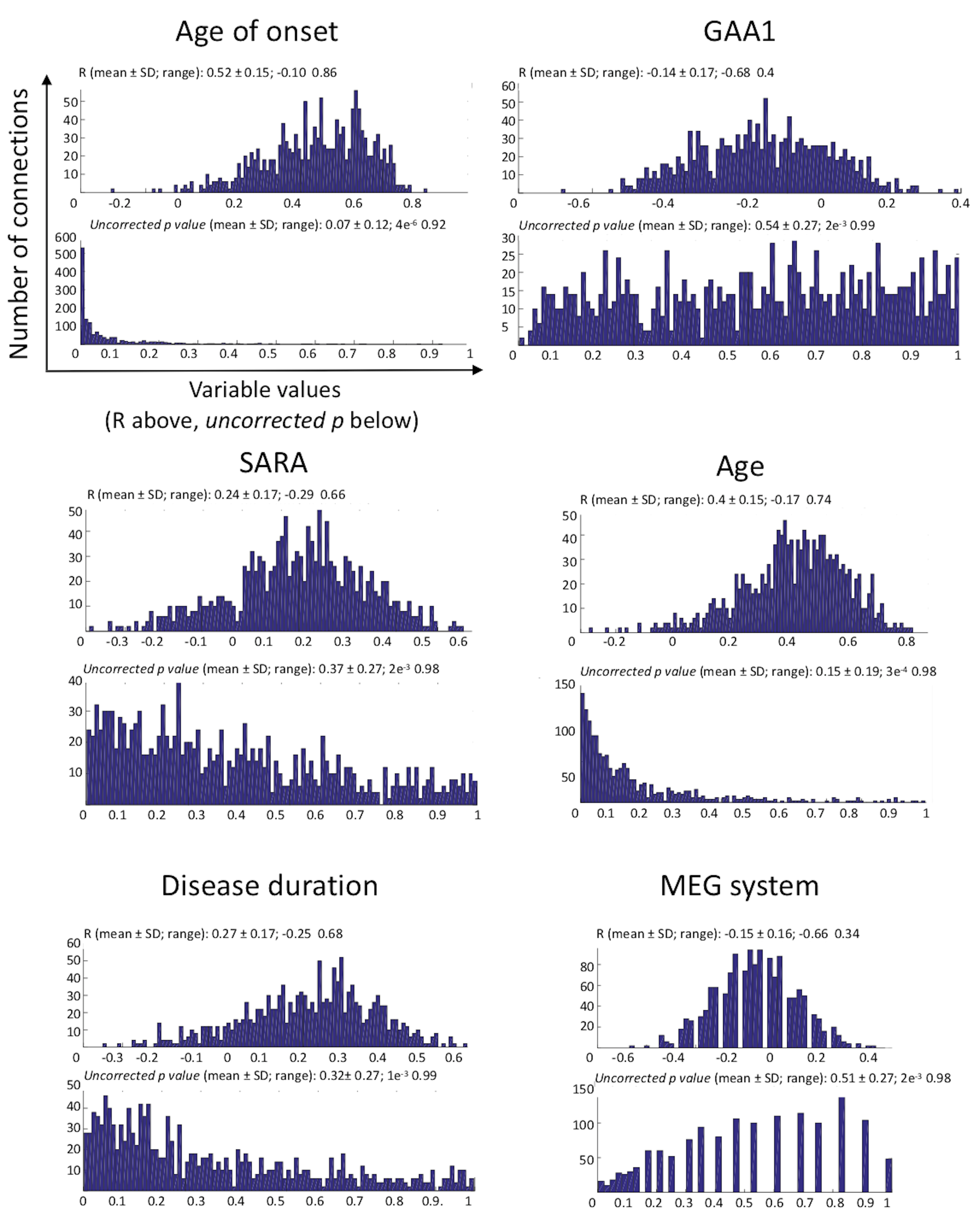

Supplement: Supplementary file 1 — Figure S1. Histograms showing the distribution of correlation coefficients and uncorrected P‐values for correlations between age of symptoms onset, the size of GAA1 triplet expansion, Age, SARA score, disease duration, and MEG system type. [file ACN3-7-94-s001.tiff]
