# Supplementary material for: Age of onset determines intrinsic functional brain architecture in Friedreich ataxia
Source: Ann Clin Transl Neurol. 2019 Dec 18;7(1):94–104. doi: 10.1002/acn3.50966 (PMC6952309; doi:10.1002/acn3.50966)
Supplement: Supplementary file 2 — Table S1. Illustrates the correlations between rsFC and MEG system, age, disease duration, GAA1, and SARA associated to its P‐value. [file ACN3-7-94-s002.docx]

**Supplementary Table 1**. Illustrates the correlations between rsFC and MEG system, age, disease duration, GAA1 and SARA associated to its *p-value*. R: correlation coefficient. Of notice, to control for multiple comparisons issues, the corrected *p-value* is the uncorrected *p-value* multiplied by the degrees of freedom (20).

| Nodes | MEG system | | Age | | Disease Duration | | GAA1 | | SARA | |
| --- | --- | --- | --- | --- | --- | --- | --- | --- | --- | --- |
|  | R | *Uncorrected p* | R | *Uncorrected p* | R | *Uncorrected p* | R | *Uncorrected p* | R | *Uncorrected p* |
| LpIPS | -0.0773 | 0.7605 | 0.5088 | 0.0311 | 0.3449 | 0.1610 | -0.1386 | 0.5958 | 0.2826 | 0.2558 |
| RpIPS | 0.0515 | 0.8391 | 0.4116 | 0.0897 | 0.1926 | 0.4437 | -0.1643 | 0.5286 | 0.1667 | 0.5086 |
| LFEF | -0.1803 | 0.4740 | 0.5233 | 0.0259 | 0.3884 | 0.1112 | -0.0822 | 0.7539 | 0.4586 | 0.0556 |
| RFEF | -0.2061 | 0.4120 | 0.5584 | 0.0160 | 0.4205 | 0.0823 | 0.0245 | 0.9256 | 0.2878 | 0.2469 |
| LMT | 0.1030 | 0.6841 | 0.2265 | 0.3662 | 0.0922 | 0.7160 | -0.1091 | 0.6767 | 0.1956 | 0.4365 |
| RMT | -0.1803 | 0.4740 | 0.4623 | 0.0534 | 0.3397 | 0.1678 | -0.2784 | 0.2793 | 0.2381 | 0.3414 |
| RMFG | -0.3091 | 0.2120 | 0.4891 | 0.0394 | 0.2745 | 0.2704 | -0.1165 | 0.6561 | 0.3489 | 0.1559 |
| RPCS | -0.1545 | 0.5403 | 0.4147 | 0.0871 | 0.2714 | 0.2760 | -0.0638 | 0.8079 | 0.2826 | 0.2558 |
| RSMG | 0.2833 | 0.2546 | 0.5471 | 0.0188 | 0.4071 | 0.0936 | -0.3519 | 0.1659 | 0.3478 | 0.1573 |
| RSTG | -0.3864 | 0.1133 | 0.4312 | 0.0740 | 0.2589 | 0.2995 | -0.2403 | 0.3528 | 0.1791 | 0.4771 |
| RVFC | 0.3348 | 0.1744 | 0.5119 | 0.0299 | 0.2279 | 0.3631 | -0.3237 | 0.2049 | 0.1708 | 0.4980 |
| LAG | 0.0515 | 0.8391 | 0.4106 | 0.0906 | 0.2175 | 0.3859 | -0.3041 | 0.2353 | 0.1925 | 0.4440 |
| RAG | 0.1803 | 0.4740 | 0.4798 | 0.0439 | 0.2859 | 0.2502 | -0.2391 | 0.3553 | 0.2795 | 0.2613 |
| PCC | -0.1030 | 0.6841 | 0.4788 | 0.0444 | 0.3698 | 0.1310 | -0.2931 | 0.2536 | 0.2329 | 0.3523 |
| vMPFC | 0.0515 | 0.8391 | 0.3009 | 0.2250 | 0.0984 | 0.6977 | -0.3838 | 0.1283 | 0.0652 | 0.7971 |
| dMPFC | -0.2576 | 0.3021 | 0.4126 | 0.0888 | 0.2227 | 0.3745 | -0.1938 | 0.4562 | 0.2381 | 0.3414 |
| RMPFC | -0.2061 | 0.4120 | 0.4095 | 0.0915 | 0.1232 | 0.6261 | -0.3335 | 0.1908 | 0.1418 | 0.5746 |
| LITG | 0.1545 | 0.5403 | 0.5657 | 0.0144 | 0.4713 | 0.0484 | -0.0809 | 0.7575 | 0.3747 | 0.1255 |
| LV1 | -0.0773 | 0.7605 | 0.5677 | 0.0140 | 0.3780 | 0.1219 | -0.3814 | 0.1310 | 0.2443 | 0.3286 |
| RV1 | -0.1288 | 0.6106 | 0.5171 | 0.0280 | 0.3035 | 0.2209 | -0.4059 | 0.1060 | 0.1925 | 0.4440 |
| LS2 | -0.1803 | 0.4740 | 0.5233 | 0.0259 | 0.3791 | 0.1208 | -0.0809 | 0.7575 | 0.3830 | 0.1167 |
| RCS | 0.2576 | 0.3021 | 0.6308 | 0.0050 | 0.4909 | 0.0386 | -0.0871 | 0.7397 | 0.4141 | 0.0876 |
| LCS | -0.1545 | 0.5403 | 0.4312 | 0.0740 | 0.3294 | 0.1820 | -0.1263 | 0.6291 | 0.3188 | 0.1972 |
| RS2 | 0.4121 | 0.0892 | 0.3475 | 0.1577 | 0.2113 | 0.4000 | -0.0650 | 0.8043 | 0.3654 | 0.1359 |
| LSMA | -0.4121 | 0.0892 | 0.6236 | 0.0057 | 0.4526 | 0.0593 | -0.0883 | 0.7361 | 0.3892 | 0.1104 |
| RSMA | 0.3348 | 0.1744 | 0.5708 | 0.0134 | 0.3967 | 0.1031 | -0.0491 | 0.8517 | 0.3737 | 0.1266 |
| LDIFG | -0.0258 | 0.9192 | 0.6494 | 0.0035 | 0.5241 | 0.0256 | -0.2673 | 0.2996 | 0.4741 | 0.0468 |
| STS | -0.0773 | 0.7605 | 0.4002 | 0.0998 | 0.3231 | 0.1909 | -0.0123 | 0.9627 | 0.4721 | 0.0479 |
| T1a | 0.1803 | 0.4740 | 0.5884 | 0.0102 | 0.5117 | 0.0300 | -0.1091 | 0.6767 | 0.3758 | 0.1243 |
| F3OPD | -0.1030 | 0.6841 | 0.5595 | 0.0158 | 0.4588 | 0.0555 | -0.2391 | 0.3553 | 0.4700 | 0.0491 |
| F3TV | -0.1545 | 0.5403 | 0.6112 | 0.0070 | 0.4816 | 0.0430 | -0.2636 | 0.3066 | 0.3934 | 0.1063 |
| T1p | -0.1545 | 0.5403 | 0.4943 | 0.0370 | 0.3314 | 0.1791 | -0.1288 | 0.6224 | 0.3302 | 0.1808 |
| DNl | 0.0515 | 0.8391 | 0.3744 | 0.1259 | 0.1378 | 0.5857 | -0.3715 | 0.1420 | 0.0983 | 0.6978 |
| DNr | -0.0515 | 0.8391 | 0.3785 | 0.1214 | 0.2165 | 0.3883 | -0.1729 | 0.5069 | 0.2195 | 0.3816 |
| cervVl | 0.1288 | 0.6106 | 0.4240 | 0.0795 | 0.2538 | 0.3096 | -0.3372 | 0.1856 | 0.0911 | 0.7192 |
| cerVr | -0.1288 | 0.6106 | 0.4954 | 0.0366 | 0.3377 | 0.1706 | -0.3458 | 0.1740 | 0.1553 | 0.5384 |
| cervVIIIl | 0.0515 | 0.8391 | 0.3961 | 0.1037 | 0.1937 | 0.4413 | -0.3948 | 0.1168 | 0.1170 | 0.6439 |
| cervVIIIr | 0.0515 | 0.8391 | 0.4126 | 0.0888 | 0.2548 | 0.3076 | -0.2575 | 0.3184 | 0.2102 | 0.4026 |
